# Towards the Abandonment of Surgical Castration in Pigs: How is Immunocastration Perceived by Italian Consumers?

**DOI:** 10.3390/ani9050198

**Published:** 2019-04-26

**Authors:** Jorgelina Di Pasquale, Eleonora Nannoni, Luca Sardi, Giulia Rubini, Renato Salvatore, Luca Bartoli, Felice Adinolfi, Giovanna Martelli

**Affiliations:** 1 Faculty of Veterinary Medicine, University of Teramo, 64100 Piano D’Accio, Teramo, Italy; jdipasquale@unite.it; 2 Department of Veterinary Medical Sciences, University of Bologna, Via Tolara di Sopra 50, 40064 Ozzano Emilia (BO), Italy; eleonora.nannoni2@unibo.it (E.N.); giulia.rubini8@unibo.it (G.R.); felice.adinolfi@unibo.it (F.A.); giovanna.martelli@unibo.it (G.M.); 3 Department of Economics and Law, University of Cassino and Southern Lazio, Viale dell’Università, 03043 Cassino (FR), Italy; rsalvatore@unicas.it (R.S.); bartoli@unicas.it (L.B.)

**Keywords:** animal welfare, consumer, willingness to pay, pig, castration, immunocastration, information, survey

## Abstract

**Simple Summary:**

The European Declaration on alternatives to surgical castration of pigs was aimed at abandoning surgical castration and switching to alternative techniques. Immunocastration (a vaccination against Gonadotropin Releasing Hormone) can be a viable alternative method. This technique offers some advantages in terms of animal welfare compared to surgical castration. Nevertheless, the main obstacle to the diffusion of immunocastration seems to be related to consumers’ acceptance, since the use of new technologies in the food chain often generates mistrust. The objective of this research was to assess how immunocastration is perceived by Italian consumers, and how complex and complete information (on advantages and disadvantages of the technique) can influence their perception. The results show that immunocastration is perceived in a predominantly positive manner (54.5%), with a relatively low level of risk perception (34.2%) and a good willingness to pay more for meat from immunocastrated pigs (+18.7%). However, there were no statistically significant differences between the control group (receiving only a neutral technical information) and groups to which complete and complex information was provided.

**Abstract:**

Immunocastration of pigs represents an alternative method to surgical castration, being more respectful of animal welfare. However, this new technology may not be accepted by consumers due to their perception of possible risks tied to the use of the product, thus representing a concern for the production sector. The study aimed at verifying the attitude of Italian consumers towards immunocastration and to assess whether their perception can be affected by science-based information on advantages and disadvantages of immunocastration. A total of 969 consumers (divided in three groups representative of the Italian population) were contacted and asked to complete an online questionnaire. Only technical (neutral) information on immunocastration was provided to the first group; the second and the third group received information on the advantages (+) and disadvantages (-) of the technique, shown in reverse order (+/- and -/+, respectively). The level of information did not affect consumers’ perception of immunocastration. Overall, immunocastration is perceived in a predominantly positive manner (54.5%), with a relatively low level of risk perception (34.2%), and a good willingness to pay more for meat deriving from immunocastrated pigs (+18.7%).

## 1. Introduction

Since 3000–4000 BC, male piglets have been surgically castrated for diverse reasons [1]: first of all, to reduce the occurrence of boar taint, which has an objectionable odor and flavor of meat deriving from entire males. The boar taint is associated with androstenone (a testicular steroid) and skatole (which is bacterially produced from tryptophan degradation in the hindgut of the pig) [2]. The second reason for castration is to reduce aggressive and sexual behaviors [3]. As a side effect of castration, it favours a higher fatness degree [4], which is appreciated for peculiar production schemes. 

At present, surgical castration is the most frequently applied method in piglets [5]. In agreement with Council Directive 2008/120/EC, this procedure is usually carried out within the first 7 days of life, with minimal (or not at all) pain relief or anesthesia. Surgical castration is an obviously painful and stressful procedure that undermines piglet’s welfare, possibly resulting in detrimental effects on growth and the immune system, and hence on the health of animals [6]. In 2010, the European Declaration on alternatives to surgical castration of pigs [7] recommended to switch to alternatives (such as castration with anesthesia or analgesia, raising entire males, sperm sexing, and immunocastration), with the aim to abandon castration by 2018, with the exception of pork meat for products under “Traditional Speciality Guaranteed -TSG”, “Protected Geographical Indication PGI” or “Protected Designation of Origin -PDO” labels, for which castration is deemed to be unavoidable to meet the current quality standards [7]. Although to date the target set by the Declaration has not been met by most European countries, the use of local anesthesia during surgical castration has been mandatory in Norway since 2002, and in Switzerland but by using general anesthesia [5].

Immunocastration is a viable alternative to surgical castration. It consists in a vaccination against GnRH (Gonadotropin Releasing Hormone), through the administration of a protein-conjugate analogue of GnRH that results in the production of antibodies against the animal’s own GnRH. This subsequently stops the synthesis of LH (Luteinizing Hormone) and FSH (Follicle Stimulating Hormone) with consequent testis regression and reduced production and accumulation of steroid hormones, including boar-taint-causing androstenone [8]. Immunocastration therefore controls boar taint and aggressive and sexual behavior [3,9]. This minimally invasive technique offers some advantages compared to surgical castration: absence of acute pain, reduced stress [10], and simplified handling (it consists of a subcutaneous injection at the base of the neck, just behind the ear). Some studies have also shown that immunocastrated pigs have a better feed conversion ratio and their carcasses have a higher percentage of lean meat than surgically castrated animals [4,5,11]. This could be due to the fact that two administrations of the vaccine are needed for a full response in pigs: the first one is aimed to prime the animals’ immune system and the second one is applied when animals approach their sexual maturity [8]. Until the second injection, the immunocastrated males are physiologically more similar to entire males than to surgically castrated animals, with consequently higher lean meat yield, potentially lower environmental impact, and higher cost efficiency [2,12,13,14]. Other studies found no differences in terms of carcasses and meat quality [1].

According to the recommendations for vaccine administration, the first injection should be given at week 17–18 or earlier, while the second should be administered at 21–22 weeks old if the pigs are slaughtered at 26 weeks old [15]. For pigs slaughtered at higher age and body weight (such as Italian heavy pigs intended for PDO dry-cured hams), a third dose becomes necessary to control boar taint [8].

The drawbacks of immunocastration are tied to the possibly increased costs for the farmer (purchase of the product and workforce for its administration), the risk of accidental self-injection by the farm workers, and the uncertain consumer’s attitude towards meat from pharmacologically castrated animals. From the economic point of view, surgical castration with local anaesthesia could represent a less expensive option in comparison with immunocastration. On the other hand, immunocastration seems to result in better feed conversion rate which can compensate the costs of vaccination, particularly in Italy where pigs are slaughtered at a very high body weight (about 170 kg) [16]. As regards the risks for farm workers, accidental self- injection may produce similar effects in people to those seen in pigs (temporary reduction in sexual hormones and reproductive functions in both men and women, adverse effect on pregnancy), with increased risk after a second or subsequent accidental injection. Therefore, according to specifications, the vaccine must only be administered with a safety vaccinator having both a needle guard and a mechanism to prevent accidental operation of the trigger [15].

One of the issues that most concerns the primary sector seems to be related to consumer acceptance. Some studies have investigated consumer’s attitude towards immunocastration [17,18,19,20,21,22,23,24,25,26]. These surveys identified two main aspects: concerns for food safety and sensitivity towards animal welfare. Based on their attitudes towards these topics, EU consumers can be divided into two main groups: one broadly in favor and other one broadly against immunocastration [17]. Across the mentioned studies, participants expressed mainly favorable attitudes towards the abandonment of castration without anesthesia and analgesia and its substitution with alternative methods, although in some cases they expressed some apprehensions about immunocastration [18,26]. In a Norwegian study, despite their considerable trust in national control authorities, respondents were skeptical towards immunocastration, due to concerns about possible residuals in meat and unpredictable long-term consequences for consumers’ health. On the other hand, these consumers categorically refused surgical castration without anaesthesia [18]. Similarly, a study carried out in Italy confirms consumers’ skepticism about the use of immunocastration in pigs intended for the production of traditional products (PDO and PGI) with doubts similar to those indicated by Norwegians [17]. Only few studies focus on the role that information plays on the consumer’s acceptance of immunocastration. The field of information is a topic of extreme importance for the primary sector. The way and the type of information provided to the consumer can result in the acceptance or refusal of an innovative production technique. The study carried out by Vanhonacker et al. [5], concludes that information concerning the potential benefits and risks of immunocastration does not affect much consumers’ attitudes. Tuyttens et al. [20], stressed the role of information type: audiovisual information revealed a more marked effect than basic and detailed written information: students were more in favor of immunocastration after viewing videos showing the different methods of castration. Consumers’ attitudes towards immunocastration change across countries, differs between citizens and stakeholders, and between different stakeholder categories (individuals vs. organizations). For example, scientists tend to consider immunocastration more favorably than producers, which tend to express worries about operator safety and public acceptance [19], this latter one in particular when the PDO/PGI supply chains are involved [17]. An exhaustive list of concerns expressed by the stakeholders involved in the pork chains across Europe is detailed in the final report of the CASTRUM project [27]. With respect to studies on consumers’ acceptance, a common limitation is the very little knowledge about boar taint, castration of male piglets, or alternative strategies to reduce the occurrence of boar taint [20]. Therefore, in order to study consumer’s attitude and perception, many variables such as education, social background, gender and age of the respondent need to be accounted for.

Despite the increasing attention toward animal welfare, studies carried out on consumers led to conflicting results on their WTP (Willingness To Pay) for immunocastration: a study carried out in 10 countries in 2013 [28] estimated the WTP in 0.04€/kg of pig meat. Vanhonacker et al. [5] examined Belgian consumers and found a 5% WTP, despite a very positive attitude towards immunocastration. Heid and Hamm [29] found among German consumers a negative WTP for immunocastrated pork compared to both castration with pain relief and fattening of entire males, but a positive WTP (+12%) for immunocastrated pork compared with castration without pain relief, as the result of the fact that all the alternatives have (perceived) drawbacks that force consumers to make trade-offs among different aspects. Lagerkvist et al. [30] found WTP for immunocastration to be 21% higher than that for surgical castration among Swedish consumers, who perceived immunocastration to be a socially viable alternative to castration without anaesthesia.

Given the Italian scenario (castration is necessary because pigs are slaughtered at a very high body weight, i.e., after sexual maturity, and they are intended for PDO, i.e., high quality, products), and considering the advantages of immunocastration in terms of animal welfare, the aim of the present study is to assess the attitude of Italian consumers towards immunocastration, and how this attitude is influenced by the extent in the detail of the information, and by the order in which information is provided.

## 2. Materials and Methods

### 2.1. Questionnaire and Consumers Sample

A questionnaire was formulated and submitted to a sample of 969 Italian consumers (supplemental material). The survey was carried out in Italy, between December 2018 and January 2019. Interviewees were contacted by a specialized agency (DemetraOpinioni.net S.r.l., Venice, Italy), with CAWI (Computer Assisted Web Interview) methodology. Participation quotas were identified in order to obtain three representative samples of the Italian population for gender, age (over 18 years), and geographic area (Northwestern, Northeastern, Center, South, Islands) [31]. People below 18 years of age, people who do not consume swine meat (or cured products), and people exceeding quotas were excluded from the survey. A total of 1463 invitations were sent and 1062 answers to the survey were received. Of these, 56 interviews were screened-out (people excluded from the survey because they were vegans, vegetarians or non-consumers of pork). The size of the remaining sample was 1006 consumers. However, after a quality control, 37 interviews were excluded from the sample (e.g., incomplete forms, partial answers). The final sample was of 969 Italian respondents. The average completion time was 15 min.

After filling the socio-demographic section (gender, age, occupation, education, household size and income, area of residence: rural vs. urban), all consumers were asked to respond to a ***first part*** of the questionnaire (14 questions) focusing on:

- consumer background (meat consumption habits, direct visual experience through visits of animal farms, attitude and perception towards the welfare of farmed animals);
- consumer knowledge (on animal-friendly foods, on swine castration).

Consumers were then asked to read attentively a short paragraph (approximately 12 lines) containing general information on the reasons why pigs are castrated, on how this procedure is at present carried out mainly surgically, and on immunocastration as a possible alternative to surgical castration. The paragraph contained technically neutral information. The information was preliminarily evaluated by a group of experts in the field of swine science and pre-tested on a small group of consumers (15 people), and was therefore modified in order to eliminate all the words that could bias the perception of the interviewees. For example, words unfamiliar to the general public or which may generate a greater sensitivity (e.g., “piglet”) have been accurately avoided, together with words suggesting advantages or disadvantages of one technique respect to the other.

All consumers were then asked to express their level of agreement with:

- the need to abandon surgical castration without pain relief and/or anaesthesia;
- the use of immunocastration.

For the ***second part*** of the questionnaire, the three groups of consumers previously identified were asked to answer to three different questionnaires, in order to study whether the information provided could affect their attitude towards immunocastration, their WTP and propensity to consume pork obtained with this technique and their risk perception.

The three groups filled the questionnaire as follows:

1. “Neutral information” group (N) (n = 319). This group, after reading the general paragraph described above, was asked to answer a short group of questions (8) on:
  - their preference in purchasing pork obtained either with the different methods of castration (surgical; surgical with analgesia and/or anesthesia, immunocastration, or meat from entire pigs or from pigs selected—genetic improvement—for low boar taint);
  - their WTP a premium price for these products;
  - their perceived risk with respect to immunocastration.
2. “Positive-negative information” group (+/-) (n = 323). This group, immediately after the first part of the questionnaire, was asked to read attentively a short paragraph on the advantages of immunocastration (“positive information”) in comparison with surgical castration (reduction in animal pain and discomfort, absence of negative effects on meat quality together with an improved feed efficiency in some cases). Immediately after, these consumers were asked to read a short paragraph (“negative information”) on the disadvantages of immunocastration (increased production costs and accidental self-injection risks for farm workers). Lastly, they were asked to respond to a set of questions (11) on
  - their preference in purchasing pork obtained either with the different methods of castration (surgical; surgical with analgesia and/or anesthesia, immunocastration, or meat from entire pigs or from pigs selected—genetic improvement—for low boar taint);
  - their WTP a premium price for these products;
  - their perceived risk with respect to immunocastration.
3. “Negative-positive information” group (-/+) (n = 327). This group was presented with exactly the same information and questions than the +/- group, but information was presented in reverse order (first the disadvantages and then the advantages of immunocastration).

The reverse order of presentation of the +/- information to these latter two groups was aimed to avoid any influence due to the order of presentation itself.

Given that the additional information was provided in two subsequent steps, positive/negative or vice versa, we defined this information as “complex”. Overall information consumers of groups 2 and 3 owned by the end of the questionnaire (neutral and positive/negative, regardless of the order in which this latter was received) is defined as “complete information”.

### 2.2. Statistical Analysis

Statistical analysis was carried out using SPSS software (v. 25.0). The dataset was organized in three groups according to the sampling methodology and the kind of information provided to consumers, and a one-way ANOVA was used. In the “neutral information” group, the perception of immunocastration was tested after providing technically neutral information, whereas in the other two groups the difference in perception was tested after providing technically neutral information and after providing the complex and complete information. Similarly, ANOVA was applied to the variables related to the willingness to consume products obtained with the use of immunocastration, the WTP and the risk perception.

Brown and Forsythe [32] and the Welch [33] tests were carried out. Using absolute deviations from the group medians, the Brown–Forsythe is a robust test for data that potentially violate the assumption of normality. The test compares the variance within each group with the median value of the variance across groups. The Welch test is an alternative test for the one factor analysis of variance F-test. It is a parametric test for equal population means, to be used when we do not have equal population variances.

Moreover, pairwise comparisons, by using Tukey HSD [34], Duncan [35], and Scheffe [36] post hoc tests, were carried out to confirm the absence of significant differences between all possible pairs of averages. The Tukey test is a non-parametric test which in our case is particularly suitable since it is structured for data measured in ordinal scales. To confirm the validity of the test, two other post hoc tests were run: the Duncan test (commonly used in agronomy and in other agricultural economics research), which is more protective against the type II error (although it implies a greater risk of type I errors), and the Scheffe test, which is a more flexible test and was used only as confirmation and reinforcement of the goodness of the ANOVA analysis.

Statistical significance was set at *p* < 0.05 for all tests.

## 3. Results

### 3.1. Consumers Background and Knowledge On Animal Welfare

According to our results, the direct knowledge of Italian consumers regarding animal welfare can be defined as very limited. Only 12.6% of the interviewees gathered their knowledge through direct visiting of farms, and for about half of them this experience was sporadic (1 or 2 farm visits in their lifetime). About one fifth (21.2%) of the responders say to have no knowledge on issues related to animal welfare and 66.3% have received their information through the mass media.

Among those who visited a farm at least once, the most common species seen is swine (n = 84), followed shortly by beef cattle (n = 79), while the less observed species is sheep (n = 4) (multiple answers allowed–total number of answers received: 411).

In general, consumers believe that avian species are those having the worst welfare conditions. On a Likert scale with a score of 1 to 5 (where 1 = minimum welfare, 5 = maximum welfare), broilers have an average score of 2.54 and laying hens 2.70. Pigs do have a level of welfare comparable to that of laying hens (2.72), while dairy cows and beef cattle get relatively higher scores (3.2 and 3.0, respectively).

On a scale from 0 to 10 (with 0 = "not at all" and 10 = "extremely"), 82% of respondents attributed a value equal or higher than 6 to the importance of animal welfare during purchases. In particular, a quarter of the interviewees stated that they attribute a valor equal to 10 to animal welfare. The average value attributed by the whole group of respondents was 8.4.

The large majority of consumers (69.7%) declared to purchase food obtained respecting a level of animal protection higher than the minimum set up by legislation; out of them, 40% declare to do it always, while the remaining 60% only sometimes. Among consumers who purchased these products, 59% bought organic foods or products obtained from animals having an outdoor access. However, only one half of those giving very high importance to animal welfare at time of purchasing (score 10 = extremely) declare to buy “animal friendly” products always.

### 3.2. Consumers Knowledge and Perception on Swine Castration

Only about a quarter of respondents (n = 259) are aware that male pigs undergo castration within the first week of their life. On a total of 381 selections made (multiple answers were allowed), 198 answers indicated that this practice is aimed to improve meat quality or meat production and 106 indicated that castration is aimed to avoid boar taint. 

After reading the short “neutral” paragraph, respondents were asked to express their level of agreement with the abandonment of surgical castration (without anesthesia and/or analgesia) in favor of alternative methods. Sixty-eight percent of respondents agreed (i.e., scores equal or above 6 on a 0-to-10 Likert scale) with the abandonment of surgical castration without anesthesia and analgesia and with the implementation of alternative castration techniques. Approximately two out of three respondents expressed a positive score (equal to or greater than 6) and more than a quarter (28%) was extremely in favor of immunocastration (scores 9 and 10).

When asked to choose among meat from surgically castrated pigs (with or without anesthesia and/or analgesia) or from animals subject to alternative methods (such as immunocastration, breeding of entire males, or animals genetically selected to not express the boar taint), consumers indicated a clear preference towards products obtained through the use of immunocastration (34%), followed equally by entire males and pigs surgically castrated with anesthesia (20.8 and 20.4%, respectively). Genetic selection was the penultimate choice (16%), followed only by surgical castration without anesthesia/analgesia.

However, at the end of the first part of the questionnaire, apprehension about immunocastration was expressed by 23% of the respondents; on the other hand, 19% had no fear of this technique, but the remaining 58% was undecided about whether the vaccine is harmless or not. Among the consumers who perceived risks or were undecided at the previous question (n = 782 consumers), 596 answers express concern about “possible unknown long-term risks”, 478 about residues in meat, and 255 indicated apprehension for pigs’ health (multiple answers allowed–total answers received: 1329).

### 3.3. Effect of Complex and Complete Information on Pig Immunocastration

Immediately after providing neutral information to all three groups, they were asked to indicate their degree of agreement with the use of immunocastration on a scale from 0 to 10. This answer will be defined as “starting point” in the results description below.

The same question was then repeated only to the second and the third groups (“positive-negative group” and “negative-positive group”) after they had received the complex and complete information. This second answer to the question will be defined as “ending point” in the results description below. It is intuitive that, for the neutral group, the starting point and the ending point coincide.

Table 1 shows the results for the question “Please indicate, on a 0-to-10 scale, your degree of agreement/disagreement with the use of immunocastration (0 = completely disagree, 10 = completely agree)”

The analysis shows no significant differences of variances among groups at the starting point. Therefore, the three groups can be considered homogeneous at the beginning of the questionnaire. Moreover, taking into account the order of the information presented, there are no significant differences at the end point after complete information has been given to the second and the third group, confirming the impossibility to reject the null hypoyhesis of equality of means. Although the sample is composed of a large number of cases, the two parametric tests have been run, assuming the possibility that the assumption of normality could be violated.

As shown in Table 2, the robust test of equality of means was carried out, with the results of Welch and Brown–Forsythe tests confirming the impossibility to reject the null hypothesis of equality of means. The post hoc tests of Tukey HSD, Duncan, and Scheffe confirmed the absence of statistically significant differences and therefore the presence of homogeneous subsets for alpha = 0.05. 

The one way-ANOVA analysis between the starting and ending point for each of the groups led to the same results as above, i.e., no significant differences were observed indicating that complex and complete information did not change consumers’ perception of immunocastration.

The results on the effect of information on the willingness to consume products obtained with the use of immunocastration, the WTP for the same products and the level of risk perception related to the technique are shown in Table 3.

Information did not affect willingness to pay or to consume products obtained from immunocastrated pigs, as confirmed also by the post hoc tests summarized in Table 4. The tests carried out confirmed the absence of significant differences between all possible pairs. A tendency (*p* = 0.063) towards a higher level of risk perception by consumers receiving at first the negative information was observed, confirmed also by the measures of Welch and Brown–Forsythe (used to confirm our results if the assumption of normality is violated) and the post hoc tests.

In absence of statistically significant differences among the three groups, they can be considered as a single representative population of Italian consumers. From our study, it emerges that average WTP stands at 18.7%, the willingness to consume products obtained through the use of immunocastration at 54.5%, and the extent to which consumers perceived the presence of risks for their health due to immunocastration was on average 34.2%.

## 4. Discussion

The results of the present survey show that the Italian population does not have a high level of direct knowledge on livestock living conditions. Most of the sample gets information on this issue from the mass media (TV, internet, and newspapers). Similar results were previously observed by our research group on a smaller sample of Italian consumers [37]. It is therefore reasonable to hypothesize that consumers’ perception of the reality of farm animals’ welfare can be chronically distorted, or at least not realistic. Information provided by media is often either entirely negative (focus on scandals, inhumane practices, mistreatments, etc.) or extremely positive (description and videos showing animals kept under bucolic conditions such as grazing on green pastures), omitting the more debatable issues/practices, e.g., mutilations.

In agreement with the findings of the 2005 Eurobarometer survey on animal welfare [38], the present study confirms that Italian consumers still perceive a marked difference between species in the level of welfare attained under common farming conditions. In particular, the welfare level of avian species is believed to be worse than cattle welfare, while pig welfare is perceived as intermediate.

Results also indicate that the Italian population is not particularly aware about the practice of pig castration. However, those who are aware of pig castration are also aware of the reasons for this practice (improving meat quality and avoiding boar taint). This lack of knowledge about castration of the general public has already pointed out by several authors [20,25,26]. The majority of respondents declared to buy product obtained with high animal welfare standards (at least sometimes), and their self-assessment regarding the level of attention paid to animal welfare during purchases returns a high score (8.4 on a 0–10 scale). This result is not entirely surprising considering that in the 2016 Eurobarometer survey on animal welfare [39], 97% of EU consumers (and the same percentage of Italian consumers) declared to perceive the protection of farmed animals as an important topic; however, it should be noted that these two questions are not directly comparable. The high percent of people indicating organic products or products obtained from animals having outdoor access mirrors the positive trend of organic market and the understanding of a link between animal welfare and ethical value of food [37].

The high attention toward animal welfare is probably the reason why the majority of respondents fully agree with the abandonment of surgical castration without anesthesia. However, there is no full agreement within the population on the alternative methodology that should be used. In fact, one-third of the respondents would prefer immunocastration, but the other two-thirds expressed preferences for other methods.

The risks perceived by Italian consumes in this study (“possible unknown long-term risks”, followed by “residues of the product in meat”) are in agreement with those recorded by Fredriksen et al. [18], although Norwegian consumers indicated the fear of residuals in meat as the main reason. Nevertheless, Italian consumers declared a good willingness to pay a premium price for products obtained from immunocastrated pigs (+18.7%). In the light of specific literature, this result can be considered as particularly positive. The value is higher than those reported in other studies on animal-friendly foods [37] and on immunocastrated meat [5,29], and similar to the WTP observed among Swedish consumers [30], although in this last study WTP was measured through a choice experiment. This is of peculiar interest for the Italian market, since it has been hypothesized that for pigs slaughtered at high body weights the increase of costs associated with immunocastration would be offset by the increase in production [16,19] and therefore would not affect the final cost of the product. It should nonetheless be noted that all studies (including the present one) based on a declared (i.e., hypothetical) WTP might lead to its overestimation.

Also, the willingness to consume pork from immunocastrated animals was positive (54.5%), even if our result is only slightly higher than the central value. This outcome must be read in light of the results obtained on the level of risk perception, i.e., consumers are not completely convinced of the harmlessness of immunocastration, even if the level of risk perception expressed by consumers was relatively low on average (34%).

The values of willingness to pay, willingness to consume, and level of risk perception do not change after reading neutral or complete and complex information. Given the lack of direct knowledge of the consumer regarding the conditions of rearing and the technique of surgical castration without anesthesia, this remains a topic to be investigated further in future research. Some studies reported similar findings [5,20] and pointed out that it is likely that the written information does not have particularly evident effects, whereas images (videos, pictures, etc.) may produce a greater impact. However, it is also possible that, given the substantial naiveness of the general public with respect to this topic, simply providing consumers with the technically neutral information about castration might have been sufficient to negatively affect their perception, by letting them know about a mutilation of which they were completely unaware before. If this is the case, it could be argued that the information we provided as “neutral” might not have been perceived as actually neutral by consumers, and every subsequent piece of information they received might not have been able to change their initial opinion. To the best of our knowledge, this aspect has never been investigated before and could be of interest for future surveys. Although the experiment did not aim to assess the effect of the information order, an aspect worth mentioning is the tendential difference we observed in the level of risk perception, giving some evidence that probably the order in which the information was provided changed at least a little the level of risk perception by consumers: in one case (+/-) it slightly decreased the level of risk perception, whereas in the other one (-/+) it slightly increased it. It would be of interest to explore if such a tendency is confirmed or becomes stronger with larger groups of consumers.

## 5. Conclusions

The sensitivity on the European population towards animal welfare is progressively increasing and the demands for adaptation of the production systems are becoming increasingly urgent. However, it is not always easy to respond to consumer demands, as the introduction of new technologies (including vaccines) in the food chain often generates mistrust. Information can shift consumers’ preference from organoleptic characteristics to intangible characteristics such as ethic attributes (e.g., animal welfare) [40]. Until a real influence of information in favor of immunocastration will be verified, the concerns of the livestock sector over the acceptance of immunocastration by consumers are obviously legitimate.

The results of this study confirm that in the Italian population the level of attention to animal welfare is increasing and that more and more consumers are looking for and buying animal-friendly products, also demonstrating their willingness to recognize a premium price to farmers for their efforts. In this study, immunocastration was perceived in a predominantly positive manner, with a relatively low level of risk perception and a good willingness to pay, showing that, once the consumer’s trust is gained (by means of transparent information on production systems), immunocastration may become an acceptable way forward for Italian producers. However, from a practical standpoint, the numerical paucity of studies concerning the adoption of immunocastration in PDO production chains may represent a gap to be filled by future research.

## Figures and Tables

**Table 1:** One-way ANOVA results for consumers’ answers to the question “Please indicate, on a 0-to-10 scale, your degree of agreement/disagreement with the use of immunocastration (0 = completely disagree, 10 = completely agree)” after receiving different levels and complexity of information.

Variables	Total	Information (Means)			ANOVA	
	Mean (n = 969)	Neutral (n = 319)	Complete +/− (n = 323)	Complete −/+ (n = 327)	F-Test	*p*-Value
Starting Point	6.18	6.40	6.14	6.00	1.321	0.267
Ending Point	6.36		6.31	6.38	0.09	0.914

**Table 2:** Robust and post hoc tests and results for consumers’ agreement/disagreement on the use of immunocastration.

		Statistic ^a^	Significance.
Starting Point	Welch	1.348	0.260
	Brown-Forsythe	1.322	0.267
Ending Point	Welch	0.092	0.912
	Brown-Forsythe	0.090	0.914
		**Starting Point**	**Ending Point**
Tukey HSD ^b,c^	Significance	0.243	0.913
Duncan ^b,c^	Significance	0.130	0.705
Scheffe ^b,c^	Significance	0.275	0.920

Means for groups in homogeneous subsets are displayed. ^a.^ Asymptotically F distributed; ^b.^ Uses Harmonic Mean Sample Size = 322,967; ^c.^ The group sizes are unequal, the harmonic mean of the group sizes is used. Type I error levels are not guaranteed.

**Table 3:** One-way ANOVA results for consumers’ willingness to consume, willingness to pay, and level of perception of risks for consumers’ health tied to immunocastration (answers were expressed on a 0-to-100 scale) after receiving different levels and complexity of information.

Variables	Total	Information (Means)			ANOVA	
	Mean (n = 969)	Neutral (n = 319)	Complete +/− (n = 323)	Complete −/+ (n = 327)	F-Test	*p*-Value
Willingness to consume	54.54	54.80	54.29	54.54	0.020	0.980
Willingness to pay	18.74	18.04	18.58	19.58	0.489	0.613
Level of risk perception	34.23	33.273	32.31	37.06	2.775	0.063

**Table 4:** Robust tests and post hoc tests results for consumers’ willingness to pay, willingness to consume, and perception of risks tied to immunocastration.

		Statistic ^a^		Significance	
Willingness to consume	Welch	0.020		0.981	
	Brown–Forsythe	0.020		0.980	
Willingnes to pay	Welch	0.490		0.613	
	Brown–Forsythe	0.490		0.613	
Level of risk perception	Welch	2.747		0.065	
	Brown-Forsythe	2.776		0.063	
		**Willingness to consume**	**Willingness to pay**		**Level of risk perception**
Tukey HSD ^b,c^	Significance	0.978	0.594		0.068
Duncan ^b,c^	Significance	0.852	0.363		0.077
Scheffe ^b,c^	Significance	0.980	0.623		0.085

Means for groups in homogeneous subsets are displayed. ^a.^ Asymptotically F distributed; ^b.^ Uses Harmonic Mean Sample Size = 322,967; ^c^ The group sizes are unequal, the harmonic mean of the group sizes is used. Type I error levels are not guaranteed.

## Supplementary Materials

The supplementary materials are available online at https://www.mdpi.com/2076-2615/9/5/198/s1.
