# Supplementary material for: Towards the Abandonment of Surgical Castration in Pigs: How is Immunocastration Perceived by Italian Consumers?
Source: Animals (Basel). 2019 Apr 26;9(5):198. doi: 10.3390/ani9050198 (PMC6562759; doi:10.3390/ani9050198)
Supplement: Supplementary file 1 [file animals-09-00198-s001.pdf]

SOCIO-DEMOGRAPHIC SECTION

(To allow the creation of representative groups of consumers)

Sex

- Female
- Male

Year of birth

____

Household size (including yourself)

____

Education

- None
- Primary school
- Middle School
- High School
- Bachelor of Science
- Master of Science

Place of residence:

____

Occupation

- Employee
- Business owner / freelance
- Retired
- Housewife
- Unoccupied
- Student
- Other

Household income per year

- Below 10.000 €
- Between 11.000 and 20.000 €
- Between 21.000 and 35.000 €
- Between 36.000 and 50.000 €
- Between 51.000 and 75.000
- Over 75.000

Urban area:

- Yes
- No

PART ONE

1) Which kind of diet do you follow?

a) Omnivorous (consumption of both vegetal products and animal-derived foods: meat, milk, eggs, etc.)
b) Vegetarian (the questionnaire ends here)
c) Vegan (the questionnaire ends here)

2) How often do you eat the following kind of meat / meat products? (e.g., salami, hamburgers, et.)?

	At least once a week	More than once a week	At least once a month	Less than once a month	Never (the questionnaire ends here)
Chicken / Turkey	○	○	○	○	○
Pork	○	○	○	○	○
Beef	○	○	○	○	○

3) Where does your knowledge on the conditions under which farm animals are raised come from?

a) I don’t have any specific knowledge
b) mass media (Television, web, newspapers)
c) direct knowledge (1-2 visits to animal farms)
d) direct knowledge (more than 2 visits to farms)
e) I am a farmer / veterinarian / agronomist / etc.
f) Other (please specify)___________

4) If you answered c, d or e, could you please indicate the species? (multiple choice)

a) Laying hens
b) Broilers / Poultry
c) Pigs
d) Beef cattle
e) Dairy cows
f) Fish
g) Other (please specify)_________

5) Have you ever heard/read about animal welfare?

a) Yes
b) No

6) Which ones, among these aspects, are the most important in determining the level of animal welfare? (multiple choice)

a) Sufficient space
b) Adequate transports
c) Quality of staff
d) Access outside
e) Natural behaviour

f) Humane slaughtering
g) Absence of mutilations
h) All of the above
i) None of the above

7) Which one/ones among the following production phases are regulated by laws on animal protection? (multiple choice)

a) Rearing
b) Feeding
c) Transport (between farms, from farm to slaughter)
d) Slaughtering
e) All of the above
f) None of the above

8) Please evaluate on a 1-to-5 scale (where 1=minimum welfare; 5= maximum welfare) the level of welfare of the following species on farm

	1	2	3	4	5
Laying hens	○	○	○	○	○
Broilers	○	○	○	○	○
Pigs	○	○	○	○	○
Beef cattle	○	○	○	○	○
Dairy cows	○	○	○	○	○
Fish	○	○	○	○	○
Other (please specify)	○	○	○	○	○

9) What products are obtained respecting high animal welfare standards? (multiple choice)

1. Organic products
2. Private labels certifying high animal welfare schemes
3. Products certified as PDO / PGI
4. Products obtained from extensive farming
5. Barn eggs
6. Biodynamic products
7. Antibiotic-free products
8. Other (please specify)

10) Please score, on a 1-to-10 scale, the importance you attribute to animal welfare at time of food purchasing

0	1	2	3	4	5	6	7	8	9	10
Not important at all										Extremely important

11) **According to your purchasing habits, do you buy products obtained respecting animal welfare standards above the minimum set up by legislation?**
   a. No
   b. Sometimes
   c. Yes (please specify which kind of product________________________________)

12) **Please indicate the degree of agreement/disagreement with each of the following statements:**
   Products obtained respecting high animal welfare standards are also… (0= completely disagree; 5= completely agree)

	0	1	2	3	4	5
More safe	○	○	○	○	○	○
More reliable	○	○	○	○	○	○
More tasty	○	○	○	○	○	○
Healthier	○	○	○	○	○	○
More fresh	○	○	○	○	○	○
Greener	○	○	○	○	○	○
More expensive	○	○	○	○	○	○
More ethical	○	○	○	○	○	○
Other (please specify______________)	○	○	○	○	○	○

13) **Are you aware that male pigs are castrated within the first week of life?**
   - ☐ Yes
   - ☐ No

14) **If you answered yes, do you know the reason/reasons why male pigs are castrated? (multiple choice)**
   - ☐ No, I do not know the reason
   - ☐ To avoid questionable odours/flavours in meat
   - ☐ To limit aggression/competition among animals
   - ☐ To improve animal growth
   - ☐ To avoid unwanted mating
   - ☐ All of the above
   - ☐ None of the above
   - ☐ Other (please specify ________________)

15 INTRO – Please read carefully the following paragraph before continuing answering the questionnaire

> In many countries of the world male pigs are castrated. The aim of castration is to avoid the development of objectionable meat odours (the so-called 'boar taint', whose development is due to sexual maturity) and to limit aggression and competition between animals.
> One of the most frequently used methods (also in Italy) is surgical castration. According to legislation, surgical castration can be carried out without the administration of anaesthetics and/or analgesics when done within the first week of age. After the 7th day of life, castration must be carried out with the administration of anaesthetics and analgesics.
> In Italy pigs are usually castrated before the 7th day of life.
> Some alternative castration methods have been proposed. Among these, the most frequently used in countries such as Brazil, New Zealand and Australia is immunocastration. This method consists in the administration of a vaccine which stops the production of sexual hormones, therefore preventing sexual maturity.

15) Do you agree with the abandonment of surgical castration without anaesthetics/analgesics and with the adoption of immunocastration?

- ☐ Yes
- ☐ No

15 NEW –Please indicate, on a 0-to-10 scale, your degree of agreement/disagreement with the use of immunocastration

0	1	2	3	4	5	6	7	8	9	10
Completely disagree										Completely agree

“NEUTRAL INFORMATION” GROUP

17 Which of the following meat products would you buy? (single choice)

- ☐ Pork from animals surgically castrated
- ☐ Pork from animals surgically castrated with the administration of anesthesia/analgesia
- ☐ Pork from animals genetically selected for their low risk of developing boar taint
- ☐ Pork from immunocastrated animals
- ☐ Pork from entire (non castrated) animals (I do not care of boar taint)
- ☐ Other (please, specify ______________________)

18 Would you be willing to pay a premium price for these products?

- ☐ Yes
- ☐ No

19 If you answered ‘yes’, which premium price would you be willing to pay? (Please express it as a percentage – your answer can exceed 100%)

_____________________%

NEW 02- Assuming that the abandonment of surgical castration and the adoption of immunocastration would improve pig welfare, at what extent, would you be willing to consume products obtained through the use of immunocastration? Please rate your score on a 0 to 100 scale.

____

New 01 – Which premium price, expressed as a percentage, would you be willing to pay for immunocastrated pork? Please rate your score on a 0 to 100 scale.

____

16 Do you think that immunocastration might carry some risks?

- ☐ Yes
- ☐ I don’t know
- ☐ No

New 03 – Please indicate (expressing it as a percentage) at what extent do you think that immunocastration might carry some risks (even if still unknown) for consumers’ health

_______________ %

16s If you answered “yes” or “I don’t know” to question 16, please indicate what kind of risks are you worried about

- ☐ Vaccine residues in meat
- ☐ Risks for pigs’ health
- ☐ Long-term, still unknown risks for the consumers
- ☐ Other (please specify__________)

END OF THE “NEUTRAL INFORMATION” GROUP

“POSITIVE-NEGATIVE INFORMATION” (+/-) GROUP

d.20 – Please read carefully the following paragraph before continuing answering the questionnaire

> When compared to surgical castration, immunocastration has some **advantages**:
> - Improvement of animal welfare: immunocastration involves 2-3 administrations of the vaccine (subcutaneous injection). The administration causes only minimal pain, especially if compared to surgical castration carried out without anesthesia/analgesia.
> - Meat quality: immunocastration does not affect meat quality. According to some studies, improvements in pigs’ productive performances can be observed.

Please indicate, on a 0-to-10 scale, your degree of agreement/disagreement with the use of immunocastration

0	1	2	3	4	5	6	7	8	9	10
Completely disagree										Completely agree

17 Which of the following meat products would you buy? (single choice)

- ☐ Pork from animals surgically castrated
- ☐ Pork from animals surgically castrated with the administration of anesthesia/analgesia
- ☐ Pork from animals genetically selected for their low risk of developing boar taint
- ☐ Pork from immunocastrated animals
- ☐ Pork from entire (non castrated) animals (I do not care of boar taint)
- ☐ Other (please, specify ______________________)

18 Would you be willing to pay a premium price for these products?

- ☐ Yes
- ☐ No

19 If you answered ‘yes’, which premium price would you be willing to pay? (Please express it as a percentage – your answer can exceed 100%)

______________________%

d21 Please read carefully the following paragraph before continuing answering the questionnaire

> When compared to surgical castration, immunocastration brings some **disadvantages:**
> - Increased costs: due to both vaccine price and manpower for vaccine administration (each male pig must receive 2-3 injections during his lifetime).
> - Risks for the operator: heavy pigs need 3 administrations of the product and the last doses must be administered when animals have reached a high body weight (100-160 kg). In case of accidental self-injection, the operator might experience similar effects to those seen in pigs, including a temporary reduction in sexual hormones and reproductive functions.

Please indicate, on a 0-to-10 scale, your degree of agreement/disagreement with the use of immunocastration

0	1	2	3	4	5	6	7	8	9	10
Completely disagree										Completely agree

D22b Do you believe that the use of immunocastration might carry additional risks, with respect to those you just read about?

- ☐ Yes (goes to question 22bb)
- ☐ No (goes to question 23)

D22bb Please indicate which kind of risks are you worried about

- ☐ Vaccine residues in meat
- ☐ Risks for pigs' health
- ☐ Long-term, still unknown risks for the consumers
- ☐ Other (please specify__________)

23 Which of the following meat products would you buy? (single choice)

- ☐ Pork from animals surgically castrated
- ☐ Pork from animals surgically castrated with the administration of anesthesia/analgesia
- ☐ Pork from animals genetically selected for their low risk of developing boar taint
- ☐ Pork from immunocastrated animals
- ☐ Pork from entire (non castrated) animals (I do not care of boar taint)
- ☐ Other (please, specify ______________________)

24 Would you be willing to pay a premium price for these products?

- ☐ Yes
- ☐ No

25 If you answered 'yes', which premium price would you be willing to pay? (Please express it as a percentage – your answer can exceed 100%)

______________________%

NEW 02- Assuming that the abandonment of surgical castration and the adoption of immunocastration would improve pig welfare, at what extent would you be willing to consume products obtained through the use of immunocastration? Please rate your score on a 0 to 100 scale.

____

New 01 – Which premium price, expressed as a percentage, would you be willing to pay for immunocastrated pork? ________________________ %

16 Do you believe that the use of immunocastration might carry some risks?

- ☐ Yes
- ☐ I don't know
- ☐ No

New 03 – Please indicate (expressing it as a percentage) at what extent do you think that immunocastration might carry some risks (even if still unknown) for consumers' health _______________ %

16s If you answered "yes" or "I don't know" to question 16, please indicate which kind of risks are you worried about

- ☐ Vaccine residues in meat
- ☐ Risks for pigs' health
- ☐ Long-term, still unknown risks for the consumers
- ☐ Other (please specify___________)

END OF THE +/- GROUP

“NEGATIVE-POSITIVE INFORMATION” (-/+) GROUP

d21 Please read carefully the following paragraph before continuing answering the questionnaire

When compared to surgical castration, immunocastration brings some **disadvantages:**

- Increased costs: due both to vaccine price and manpower for vaccine administration (each male pig must receive 2/3 injections during his lifetime).
- Risks for the operator: heavy pigs need 3 administrations of the product and the last doses must be administered when animals have reached a high body weight (100-160 kg). In case of accidental self-injection, the operator might experience similar effects to those seen in pigs, including a temporary reduction in sexual hormones and reproductive functions.

Please indicate, on a 0-to-10 scale, your degree of agreement/disagreement with the use of immunocastration

0	1	2	3	4	5	6	7	8	9	10
Completely disagree										Completely agree

D22b Do you believe that the use of immunocastration might carry additional risks, with respect to those you just read about?

- ☐ Yes (goes to question 22bb)
- ☐ No (goes to question 23)

D22 bb please indicate which kind of risks are you worried about

- ☐ Vaccine residues in meat
- ☐ Risks for pigs’ health
- ☐ Long-term, still unknown risks for the consumers
- ☐ Other (please specify__________)

17 Which of the following meat products would you buy? (single choice)

- ☐ Pork from animals surgically castrated
- ☐ Pork from animals surgically castrated with the administration of anesthesia/analgesia
- ☐ Pork from animals genetically selected for their low risk of developing boar taint
- ☐ Pork from immunocastrated animals
- ☐ Pork from entire (non castrated) animals (I do not care of boar taint)
- ☐ Other (please, specify ______________________)

18 Would you be willing to pay a premium price for these products?

- ☐ Yes
- ☐ No

19 If you answered ‘yes’, which premium price would you be willing to pay? (Please express it as a percentage – your answer can exceed 100%)

_____________________%

d.20 Please read carefully the following paragraph before continuing answering the questionnaire

> Compared to surgical castration, immunocastration has some **advantages**:
> - Improvement of animal welfare: immunocastration consists of 2-3 administrations of the vaccine (subcutaneous injection). The administration causes only minimal pain, especially if compared to surgical castration carried out without anesthesia/analgesia.
> - Meat quality: immunocastration does not affect meat quality. According to some studies, improvements in pigs' productive performances can be observed.

Please indicate, on a 0-to-10 scale, your degree of agreement/disagreement with the use of immunocastration

0	1	2	3	4	5	6	7	8	9	10
Completely disagree										Completely agree

23 Which of the following meat products would you buy? (Single choice)

- ☐ Pork from animals surgically castrated
- ☐ Pork from animals surgically castrated with the administration of anesthesia/analgesia
- ☐ Pork from animals genetically selected for their low risk of developing boar taint
- ☐ Pork from immunocastrated animals
- ☐ Pork from entire (non castrated) animals (I do not care of boar taint)
- ☐ Other (please, specify ______________________)

24 Would you be willing to pay a premium price for these products?

- ☐ Yes
- ☐ No

25 If you answered 'yes', which premium price would you be willing to pay? (Please express it as a percentage – your answer can exceed 100%)

_____________________%

NEW 02- Assuming that the abandonment of surgical castration and the adoption of immunocastration would improve pig welfare, at what extent would you be willing to consume products obtained through the use of immunocastration? Please rate your score on a 0 to 100 scale.

____

New 01 – Which premium price, expressed as a percentage, would you be willing to pay for immunocastrated pork? ________________________ %

16 Do you believe that the use of immunocastration might carry some risks?

- ☐ Yes
- ☐ I don't know
- ☐ No

New 03 – Please indicate (expressing it as a percentage) at what extent do you think that immunocastration might carry some risks (even if still unknown) for consumers' health _______________ %

16s If you answered "yes" or "I don't know" to question 16, please indicate which kind of risks are you worried about

- ☐ Vaccine residues in meat
- ☐ Risks for pigs' health
- ☐ Long-term, still unknown risks for the consumers
- ☐ Other (please specify__________)

END OF THE -/+ GROUP
